# Assimilation of Phosphate of Lime and Its Therapeutical Employment

**Published:** 1869-06

**Authors:** 


Assimilation of Phosphate of Lime and its Therapeutical Employment.—MM. Dusart and Blache, of Paris, have endeavored to determine the question whether the phosphate of lime enters into the system by the transformations it undergoes in the stomach, or whether it is necessary, for the purpose of assimilation, that it should undergo a previous elaboration in a living organism. The experiments they have instituted appear to show that the solution of the phosphate in the juices of the stomach is influenced by the form which the phosphate assumes, for while the hydrated phosphate is rapidly dissolved, calcined bones and hartshorn are not sensibly dissolved; and specimens containing carbonate of lime are dissolved only imperfectly. Messrs. Dusart and Blache, therefore, propose, as the best preparation for assimilation, the hydrated phosphate which has already been subjected to the action of the gastric acids, and which they call *lacto-phosphate of lime.* This substance has an agreeably acidulous taste, and is readily digested. Experiments were made upon some of the lower animals, with a view of determining whether the repair of fractured bones was accelerated by the internal use of the phosphate, and it was found that such was really the result. Under the use of the *lacto phosphate of lime*, Messrs. Dusart and Blache, found that the increase in weight of the bones of the animals exceeded by more than 33 per cent. the weight of the animals subjected to ordinary treatment. The animals chosen for the experiment were guinea-pigs.—*British and Foreign Med.-Chir. Rev.*

